# What is the best etching timing in a resin infiltrant used on enamel surface after remineralization with CPP-ACP?

**DOI:** 10.1080/07853890.2021.1897416

**Published:** 2021-09-28

**Authors:** Joana Carmo, Carla Ascenso, Ana Jorne, André Peixoto, Isabel Nogueira, Ana Garcia Manso

**Affiliations:** a Centro de Investigação Interdisciplinar Egas Moniz (CiiEM), Egas Moniz Cooperativa de Ensino Superior, Caparica, Portugal; b MicroLab - Electron Microscopy Laboratory of Instituto Superior Técnico - Universidade de Lisboa, Lisboa, Portugal

## Abstract

**Introduction:**

Nowadays there are non-invasive therapeutical options to treat white spot lesions (WSL), such as remineralization with CPP-ACP pastes and infiltrative technics with infiltrant resins which fill the gaps caused by demineralisation, preventing the progression of the initial lesion [1,2]. The aim of this exploratory study is to observe the enamel surface after artificial induction of caries, when a pre-treatment with Casein Phosphopeptide - Amorphous Calcium Phosphate is applied, followed by an enamel infiltrating resin, with different times of acid conditioning.

**Material and methods:**

6 specimens were obtained from 2 human molars and randomly distributed into 3 groups (A, B and C). All the groups undergo artificial white spot induction through lactic acid buffer solution for 6 days, and CPP-ACP treatment for 4 weeks. Group A remained without any further treatment; B and C were treated with infiltrating resins with different acid conditioning timings, 2 and 3 min respectively. After treatment, 2 specimens of each group were visualised by scanning electron microscopy (SEM).

**Discussion and results:**

According to the SEM analysis, this exploratory study suggests that group A is more homogeneous, with less exposure of gaps compared to groups B and C, which are more heterogeneous, C more than B, as demonstrated in Image 1.

**Conclusions:**

Group A treated with CPP-ACP presents the most homogenous images of enamel surface. The images of group C, treated with 3 min of acid conditioning suggest a greater demineralisation than images of group B (2 min).

**Image 1.**
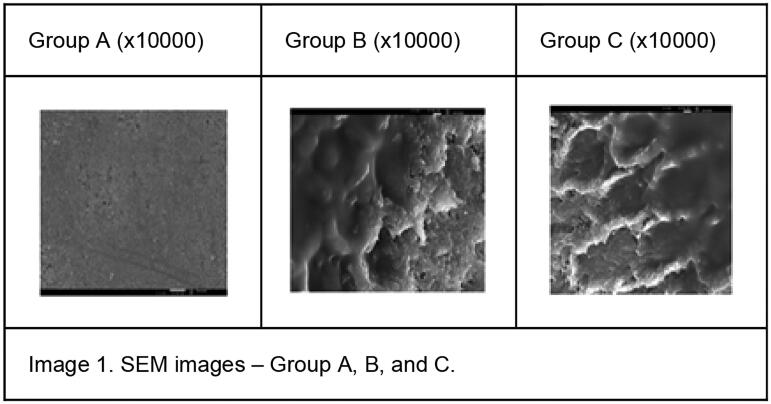
SEM images – Group A, B, and C.
